# Effect of a transactional model education program on coping effectiveness in women with multiple sclerosis

**DOI:** 10.1002/brb3.810

**Published:** 2017-09-21

**Authors:** Hormoz Sanaeinasab, Mohsen Saffari, Mahrokh Hashempour, Ali‐Akbar Karimi Zarchi, Waleed A. Alghamdi, Harold G. Koenig

**Affiliations:** ^1^ Health Education Department Faculty of Health Baqiyatallah University of Medical Sciences Tehran Iran; ^2^ Health Research Center Baqiyatallah University of Medical Sciences Tehran Iran; ^3^ Department of Epidemiology and Biostatistics Faculty of Health Baqiyatallah University of Medical Sciences Tehran Iran; ^4^ Department of Psychiatry King Abdul Aziz University Jeddah Saudi Arabia; ^5^ Department of Psychiatry and Behavioral Sciences Duke University Medical Center Durham NC USA; ^6^ King Abdulaziz University Jeddah Saudi Arabia; ^7^ Ningxia Medical University Yinchuan China

**Keywords:** coping, education, multiple sclerosis, stress, transactional model

## Abstract

**Objectives:**

Multiple sclerosis (MS) is a chronic and progressive disease that causes stress due to its unpredictability and lack of definitive treatments. This study examined the effects of an educational program using a transactional model to help women with MS cope with their disease.

**Materials and Methods:**

In a randomized clinical trial, 80 female patients from the MS Society of Iran were randomized to the intervention (*n* = 40) or a control group (*n* = 40). Outcomes were assessed using Cohen's Perceived Stress Scale (PSS) and the Jalowiec Coping Scale (JCS), which were completed by both groups at baseline, 1 month, and 3 months after the intervention. The intervention consisted of six educational sessions administered over 2 months based on a transactional model. The data were analyzed using repeated measures ANOVA.

**Results:**

Average PSS scores decreased significantly over time in the intervention group, while increasing in the control group. Between‐group differences were significant at both 1‐month and 3‐month follow‐up (*p* < .001). Both problem‐focused and emotion‐focused coping styles improved over time in use and effectiveness in the intervention group, whereas little or no change occurred in these coping behaviors in the control group.

**Conclusion:**

The transactional model‐based education program tested here was successful in reducing stress levels and increasing healthy coping styles in women with MS. If these findings are replicated in future studies, widespread adoption of this program may help women with MS cope more successfully with their disease.

## 1 INTRODUCTION

Multiple sclerosis (MS) is a chronic neurological disease characterized by many distressing clinical symptoms, such as sensorimotor deficits, ataxia, neuropsychiatric disorders, fatigue, and dysphagia (Bergamaschi et al., 2009; Solaro et al., 2013). A wide range of complications may also be observed including uncoordinated movement, dizziness, increased heart rate and blood pressure, incontinence, and pain. MS is among the most common neurological diseases in humans and the most common debilitating disease in young adults (Cook, 2006). The disease has several debilitating effects during the early stages such as fatigue and cognitive impairment; however, after 20 years, more than 60% of affected individuals will not be able to walk without assistance. This condition can cause serious problems for patients, family members, and friendship networks, and is extremely costly for society unless managed properly (Rodriguez, 2008). The number of people with MS increased from 2.1 million in 2008 to more than 2.3 million worldwide in 2016 (Browne et al., 2014; National MS Society, 2016). Three to seven individuals per 100,000 are diagnosed every year with MS (Thompson et al., 2008). Over 500,000 Americans live with this disease and 8,000 new cases are added every year (Noonan et al., 2010). In developing countries such as Iran, the disease has become epidemic in nature with a prevalence rate of between 5 and 80 per 100,000 per year (Eskandarieh, Heydarpour, & Sahraian, 2015). In a recent study, the annual incidence of MS in Iran was reported to be 5.9 per 100,000 people and the point prevalence in the Fars province of Iran was estimated to be 72 per 100,000 (Etemadifar et al., 2014; Izadi, Nikseresht, Poursadeghfard, Borhanihaghighi, & Heydari, 2015). The prevalence of the disease may be affected by gender, being more prevalent in women than in men (Harbo, Gold, & Tintore, 2013). Consequently, women are at higher risk of morbidity and should be considered a vulnerable population in whom the development of effective interventions is a high priority.

According to recent reports from the American Academy of Neurology, life stressors are important etiological factors for MS exacerbations. Not only are the symptoms of the disease worsened by stress, but more energy is needed to think about and solve problems during stressful events (AAN, 2016). The subsequent loss of energy leads to fatigue and impairments in daily functioning, and ultimately adversely affects quality of life (QOL). Many studies show the negative impact of stress on health status and QOL, and these effects are greater in MS patients than in healthy populations (Gruenewald, Higginson, Vivat, Edmonds, & Burman, 2004; Morales‐Gonzales, Benito‐Leon, Rivera‐Navarro, Mitchell, & Grp, 2004). Physical disabilities such as visual impairment, difficulty with mobility, and emotional disorders such as depression may intensify when MS patients have difficulty adjusting to their disease (Minden et al., 2013).

Given that MS is a degenerative and progressive disorder that diminishes ability to perform activities of daily living and has negative socioeconomic and emotional effects on the individual, family members, and society, medical therapy alone has proven insufficient, requiring psychosocial approaches to help overcome problems caused by this disease (Simpson, McLean, Guthrie, Mair, & Mercer, 2014). MS patients need to acquire greater knowledge and skills, improve their attitudes, and gain access to adequate resources for better coping and adaptation (Nowaczyk & Cierpialkowska, 2016). New healthy coping behaviors that improve self‐care and self‐sufficiency may be taught through education programs (Kopke et al., 2012). Patient education has now become a major part of healthcare personnel's job duties, and involves the communication of information on healthy lifestyles and instructions on self‐care (Potter & Perry, 2007).

Successful coping with a serious disease like MS needs to be understood in terms of an all‐encompassing underlying theory. There are many theories that serve to guide how a person copes with a stressor. Coping simply refers to the effort that one exercises to diminish or tolerate the negative effects of a stressful experience (Folkman & Lazarus, 1980). Coping methods may be classified in two general categories: trait‐oriented vs. state‐oriented and macroanalytic vs. microanalytic types. Trait‐oriented strategies focus on identifying people who do not have enough resources for coping, while state‐oriented strategies are those that emphasize the results of coping in term of efficiency, performance, or benefit to mental health. In a same way, the microanalytic approach involves the various types of coping strategies, while the macroanalytic approach refers to more general basic coping procedures (Zeidner & Endler, 1996). Lazarus and Folkman suggested a twofold categorization of coping strategies: problem‐focused and emotion‐focused coping (macroanalytic approach). However, they argued that to measure these categories, ways of coping should be identified (microanalytic strategy). In studies conducted in general populations, they found eight groups of coping strategies: confrontative, social support, distancing, self‐control, escape/avoidance, reappraisal, problem solving, and responsibility‐related copings. However, they did not definitely organize these copings into two basic categories (i.e., problem‐focused vs. emotion‐focused strategies) (Folkman & Lazarus, 1988). There are other overarching classifications of coping strategies. For instance, Weiten introduced a four‐level categorization that emphasizes appraisal, emotion, problem, and occupation‐related strategies. Another classification involves dividing coping strategies in two categories: positive or adaptive techniques vs. negative or maladaptive methods (also called “non‐coping”) (Weiten, 2009).

Lazarus and Folkman's transactional model of stress and coping (TMSC) is one method of organizing ways that people cope with chronic disease. In this model, stress is viewed as a product of the interaction between the individual and the environment. This relationship is mediated by the processes of cognitive appraisal and coping. Through appraisal, a person evaluates the level of threat of a situation. The level of stress experienced is more related to the perceived level of threat rather than the actual life situation itself. Emotion‐focused coping refers to the actions people use to manage the distress itself which include strategies such as seeking social support, relaxation, and mindfulness. Problem‐focused coping, in contrast, describes the strategies used to address the problem causing the distress such as conflict resolution or problem solving (Lazarus & Folkman, 1987). These coping strategies may or may not lead to healthy constructive outcomes. The effectiveness of coping depends on the strategy that is used. For example, some types of emotion‐focused coping such as positive thinking, and being optimistic may improve some outcomes, while strategies such as avoidance or being pessimistic may have harmful results. Only a few studies, however, have examined the efficacy of such strategies as an intervention. For example, a study conducted by Nejati et al. on coping strategies among patients with hypertension found that both problem‐focused and emotion‐focused methods of coping may be enhanced by an educational intervention for stress reduction (Nejati, Zahiroddin, Afrookhteh, Rahmani, & Hoveida, 2015). Plow et al. also found that the transactional model can help MS patients in terms of improving physical exercise habits (Plow, Resnik, & Allen, 2009). In a study conducted by de Ridder et al., optimism was identified as a disease‐specific and effective coping strategy among MS patients who did not require emotion‐oriented coping (de Ridder, Schreurs, & Bensing, 2000). Schwartz in a randomized controlled trial found that quality of life was improved more by education in coping skills than was found for a peer support group (Schwartz, 1999). Another study found that degree of impairment among MS patients was related to the amount of social support they needed for coping and that special training was necessary to enhance this coping strategy (Rommer, Suhnel, Konig, & Zettl, 2016). We could find no other theory‐/model‐based program for intervening in MS patients. As these patients need special attention in terms of coping strategies and recognition of their coping resources and demands, further studies involving educational programs are warranted. So, this study was conducted to determine the effect of a transactional model‐based education program on stress and coping in women with MS in Iran.

## 2 MATERIALS AND METHODS

### 2.1 Participants and procedures

**Figure 1:**
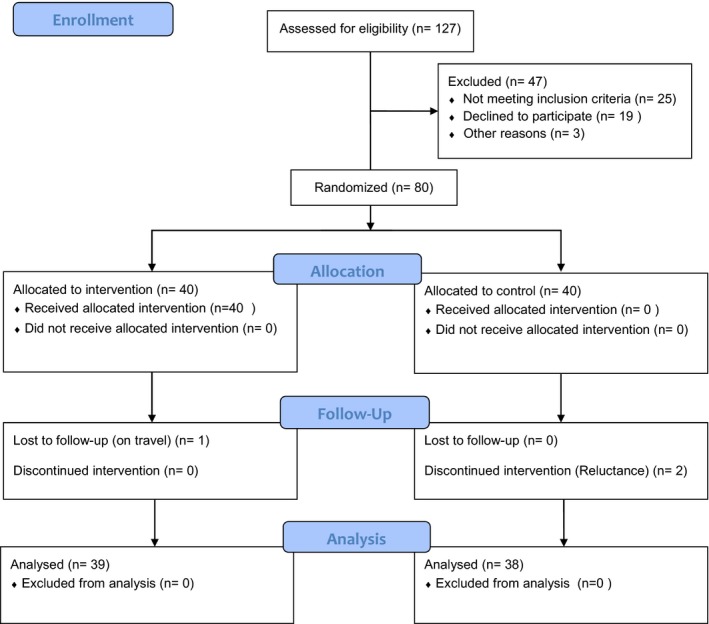
Consort Flow Diagram of the study

A randomized controlled trial was conducted in 80 women with MS patients recruited from the MS Society of Iran. We first contacted the director of this society and explained the objective of the study. With the director's permission, MS patients were identified from a list of daily visits. This was a convenience sample of those coming to the society to receive treatment. Participants were randomized either to the intervention (*n* = 40) or the control (*n* = 40) group. Study inclusion criteria were a diagnosis of MS based on McDonald's criteria (McDonald et al., 2001), at least 6 months after diagnosis of the disease, between the ages of 20 and 45, able to speak and communicate in Persian, and able to read and write. Study exclusion criteria were having other acute or chronic physical disorders that might impede the intervention, severe visual or speech impairments, and being in the acute stages of MS. After obtaining informed consent, data were collected through administration of questionnaires. Participants were randomized into intervention and control groups using block randomization. Those in the intervention group underwent 6 weekly 1‐hr training sessions based on the TMSC arranged by the Iran MS society. The educational program in the intervention group focused on problem solving, conflict resolution, mindfulness, and relaxation, which were presented to participants during group discussions, lectures, and question and answer sessions (for details see Intervention). Participants in the control group received no active intervention other than the standard psychosocial care provided by the MS Society. Questionnaires assessing stress level and coping behaviors were completed by participants in the intervention and control groups at baseline, 1 and 3 months after the intervention (process of participation in the study from enrollment to data analysis is described in Figure 1). The study protocol was approved by the ethics committee of Baqiyatallah University of Medical Sciences.

### 2.2 Measures

Age, level of education, occupation, marital status, income level, type of insurance, disease duration, and the most distressing symptom were assessed in the baseline questionnaire.

### 2.3 Perceived stress scale (PSS)

The PSS, developed by Cohen et al., asks about life stressors experienced during the past month and is based on the principle that the individual's perception of stressful life events affects health more than does the event itself. The PSS consists of 10 items and is scored on a 5‐point Likert scale (0: Never; 1: Almost Never; 2: Sometimes; 3: Fairly Often; and 4: Very Often). The score obtained on this scale ranges from a minimum of 0 to a maximum of 40, where higher scores indicate a greater degree of perceived stress. The validity and reliability of PSS have been demonstrated in Iranian samples (Maroufizadeh, Zareiyan, & Sigari, 2014).

### 2.4 Jalowiec coping scale (JSC)

The JSC consists of 60 items that assess the types of coping behaviors, and in part A, the frequency of use on a 4‐point Likert scale from 0 (never used) to 3 (often used) is asked. In part B, the respondent should identify the helpfulness of each coping strategy based on a 4‐point Likert scale from 0 (not helpful) to 3 (very helpful). The JCS was initially based on the TMSC to assess problem‐focused and emotion‐focused types of coping but there is no exact classification of coping strategies into this two categories. This scale covers eight coping styles: confrontive (10 items), evasive (13 items), optimistic (9 items), fatalistic (4 items), emotive (5 items), palliative (7 items), supportive (7 items), and self‐reliant (7 items) copings. Not all these coping strategies are adaptive and some strategies such as evasive or fatalistic may be maladaptive, while confrontive and optimistic coping strategies may be viewed as adaptive. However, there is no definite classification of these strategies into healthy/adaptive or unhealthy/maladaptive. A higher score in each domain indicates the frequency of use and its perceived effectiveness. This instrument has been previously validated in Iranian MS patients and demonstrates solid psychometric properties in this population (Saffari et al., 2016).

### 2.5 Intervention

Those in the intervention group received six 1‐hr weekly sessions of the educational program in two groups of 20. The first session included an introduction and brief review of the objectives, a discussion of the difference between feelings and thoughts, the definition of the term ‘stress,’ and a description of problem‐focused coping (PFC) and emotion‐focused coping (EFC) styles based on the transactional model, differences between these coping styles, and how they should be used in different situations. Problem‐focused strategies such as problem solving and conflict resolution, and emotion‐focused strategies such as progressive relaxation, mindfulness, and proper breathing techniques were explained. Participants were asked to perform 20‐min breathing exercises twice daily (each morning and night) as their homework assignment.

The second session consisted of a 10‐min breathing exercise, a discussion of the effects of progressive relaxation as an emotion‐focused strategy on disease symptoms, the practice of progressive relaxation, getting participants' feedback about their feelings after performing this exercise, and a description of the benefits of this practice for body and mind control. Participants were then asked to perform 20‐min progressive relaxation exercises during the week as they were assigned on the first session.

The third session consisted of a group discussion of experiences with progressive relaxation and how to deal with problems when encountered, a repetition of the 10‐min breathing exercise, and a presentation on the stages of problem solving and how to use this skill as a problem‐focused strategy for coping with daily stresses. The use of problem‐focused coping was described in seven stages: (i) identify the problem, (ii) precisely define the problem, (iii) find solutions for the problem (brainstorming), (iv) assess the solutions, (v) choose the right solution, (vi) implement the solution, and (vii) assess the problem‐solving exercises. Participants were asked to write down three problems as discussed in the session and perform 20 min of progressive relaxation during the week as their homework assignment.

The fourth session followed up on the previous session's assignment, and discussed as a group any problems with the assignment. Participants then performed the 10‐min breathing exercise, and a presentation on mindfulness as an emotion‐focused strategy. Next, a definition of the term ‘mindfulness’ was provided, followed by a discussion of the benefits of mindfulness in daily life, a presentations on how to describe stressful events and the differences between anxiety and depression. Mindfulness exercises were performed during the session, and participants were encouraged to practice it at home. Next, participants were taught how to describe a stressful life event and then score it on a scale of 0 to 10. Participants were then asked to describe three stressful life events as discussed in the session and perform 20 min of progressive relaxation twice daily during the week as before.

The fifth session consisted of an overview of the previous session's assignment and a group discussion concerning it, a 10‐min breathing exercise as before, and a presentation on conflict resolution as a problem‐focused strategy. The process of conflict resolution was described as consisting of five steps: (i) preparing for negotiation, (ii) establishing the conversation, (iii) hearing the opponent's suggestions, (iv) handling disagreement, and (v) coming to agreement. Participants each practiced one case of conflict resolution during the session and were then asked to write down another case as their assignment.

The sixth and final session consisted of an overview of the previous session's assignment and a group discussion, a review of subjects discussed in sessions 2 through 5, and a book was distributed on the subject of stress that could be reviewed to remind them of what they had learned during the sessions.

### 2.6 Statistical analyses

The data were analyzed using SPSS version 20 for Windows. To compare differences between the two groups on demographics, chi‐square and Fisher exact tests were used for categorical variables. The Student's *t* test and repeated measures analysis of variance (RMANOVA) were used for continuous variables and to compare pre‐ and postintervention scores within and between groups. Normality of the data and homoscedasticity were assessed by Kolmogrov–Smirnov and Leven tests, respectively, before conducting the RMANOVA and *t* test. The Box's test of equality of covariance matrices examined fitness of data for multivariate analysis. Interactions between time and group were also assessed. Based on Mauchly's test of sphericity, the Greenhouse‐Geisser correction was used to indicate time effect and time–group interaction. Pairwise comparisons were conducted using Bonferroni correction. The alpha level for all statistical tests was set at *p* < .05.

## 3 RESULTS

The mean ages of participants in the intervention and control groups were 29.4 (*SD*, 7.5) and 32.0 (*SD*, 5.9), respectively (Table 1). The average duration of illness for the entire group was 4.8 years (*SD*, 3.5). There were no significant differences at baseline between intervention and control groups on age, education, marital status, job status, economic status, health insurance, disease duration, or primary disease‐related problem.

**Table 1:** Characteristics of intervention and control groups

Variable	Trial	Control	*p* value
Age (year)	*N* (%)	*N* (%)	
<30	21 (52.5)	18 (45)	.654
≥30	19 (47.5)	22 (55)	
Marriage status			
Single	21 (52.5)	23 (57.5)	.822
Married	19 (47.5)	17 (42.5)	
Education			
High school	21 (52.5)	18 (45)	.654
University	19 (52.5)	22 (55)	
Job			
Housekeeper	32 (80)	30 (75)	.788
Employed	8 (20)	10 (25)	
Economic status			
Acceptable	30 (75)	28 (70)	.802
Unacceptable	10 (25)	12 (30)	
Health Insurance			
Yes	31 (77.5)	34 (85)	.566
No	9 (22.5)	6 (15)	
Disease duration			
<5	23 (57.5)	26 (65)	.646
≥5	17 (42.5)	14 (35)	
Primary disease complaint			
Weakness & fatigue	28 (70)	24 (60)	.673
Mobility deficit	4 (10)	7 (17.5)	
Visual disorder	4 (10)	6 (15)	
Others	4 (10)	3 (7.5)	

PSS scores at the three assessment points are compared in Table 2. While average PSS scores for the control group increased over time, those for the intervention group decreased over time, with between‐group differences significant at both 1‐month and 3‐month follow‐ups (both *p* < .001).

**Table 2:** A comparison of average scores on perceived stress before the intervention (Time 1), 1 month (Time 2), and 3 months (Time 3) afterward in intervention and control groups

Group	Time 1	Time 2	Time 3	*p* value (Comparison)
	Mean (*SD*)	Mean (*SD*)	Mean (*SD*)	
Trial	23.02 (6.52)	15.55 (4.77)	13.37 (3.94)	<.001 (T1 > T2 > T3)
Control	20.87 (8.62)	21.92 (7.74)	22.89 (6.49)	<.01 (T1 < T2 < T3)

T1, Time 1; T2, Time 2; T3, Time 3.

**Figure 2:**
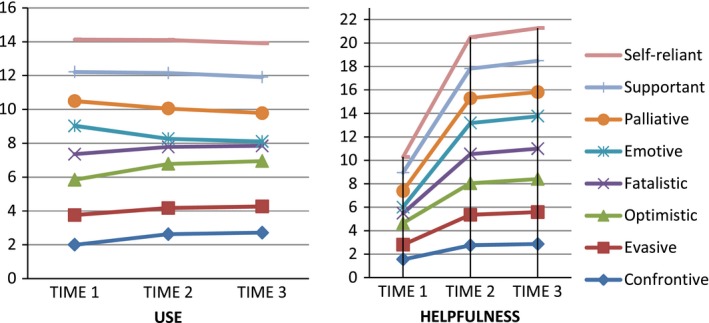
Trend of changes in the use and helpfulness of different coping style over time in the intervention group

Table 3 lists scores for each of the eight coping styles across the three assessment times in terms of use and helpfulness. Those in the intervention group increased significantly on all healthy coping styles (problem focused) in term of their use and their helpfulness (Figure 2). In contrast, most participants in the control group did not show an increase in use or helpfulness in these copying styles. Significant differences were found between intervention and control groups over time on all eight coping styles (*p* < .001).

**Table 3:** Comparison of average scores on coping style use and helpfulness at baseline (Time 1), 1‐month (Time 2), and 3‐month (Time 3) follow‐up evaluations in intervention and control groups

Coping style	Group	Part	Time 1	Time 2	Time 3	*p* value* (comparison)
			Mean (*SD*)	Mean (*SD*)	Mean (*SD*)	
Confrontive	Trial	Use	1.55 (0.60)	2.75 (0.14)	2.86 (0.11)	< .001
		Helpfulness	2.00 (0.54)	2.62 (0.17)	2.71 (0.15)	< .001
	Control	Use	1.75 (0.53)	1.66 (0.40)	1.65 (0.43)	NS
		Helpfulness	1.65 (0.56)	1.57 (0.40)	1.55 (0.44)	NS
Evasive	Trial	Use	1.75 (0.36)	1.55 (0.15)	1.55 (0.15)	< .01(T1 > T2,T3)
		Helpfulness	1.25 (0.46)	2.60 (0.18)	2.72 (0.19)	< .001
	Control	Use	1.52 (0.42)	1.52 (0.43)	1.53 (0.43)	NS
		Helpfulness	1.34 (0.45)	1.39 (0.47)	1.37 (0.50)	NS
Optimistic	Trial	Use	2.09 (0.44)	2.61 (0.17)	2.68 (0.16)	< .001
		Helpfulness	1.84 (0.60)	2.68 (0.14)	2.83 (0.11)	< .001
	Control	Use	1.93 (0.48)	1.67 (0.44)	1.68 (0.41)	< .001(T1 > T2,T3)
		Helpfulness	2.00 (0.58)	1.63 (0.46)	1.63 (0.48)	< .001(T1 > T2,T3)
Fatalistic	Trial	Use	1.51 (0.74)	1.00 (0.26)	0.90 (0.23)	< .001
		Helpfulness	0.84 (0.59)	2.50 (0.40)	2.58 (0.38)	< .001
	Control	Use	1.22 (0.57)	1.21 (0.50)	1.24 (0.46)	NS
		Helpfulness	0.95 (0.54)	1.15 (0.60)	1.16 (0.60)	< .05 (T1 < T3)
Emotive	Trial	Use	1.68 (0.67)	0.47 (0.24)	0.26 (0.16)	< .001
		Helpfulness	0.52 (0.42)	2.65 (0.32)	2.77 (0.29)	< .01
	Control	Use	1.41 (0.48)	2.12 (0.44)	2.10 (0.47)	< .001(T1 < T2,T3)
		Helpfulness	1.08 (0.47)	1.93 (0.49)	1.93 (0.48)	< .001(T1 < T2,T3)
Palliative	Trial	Use	1.47 (0.46)	1.79 (0.19)	1.68 (0.16)	< .05
		Helpfulness	1.38 (0.53)	2.11 (0.19)	2.06 (0.13)	< .001(T1 < T2,T3)
	Control	Use	1.74 (0.46)	1.98 (0.48)	1.97 (0.49)	< .01(T1 < T2,T3)
		Helpfulness	1.38 (0.52)	1.48 (0.52)	1.51 (0.47)	NS
Supportive	Trial	Use	1.72 (0.55)	2.10 (0.20)	2.12 (0.19)	< .001(T1 > T2,T3)
		Helpfulness	1.56 (0.59)	2.53 (0.31)	2.66 (0.27)	< .01
	Control	Use	1.66 (0.48)	1.69 (0.58)	1.70 (0.57)	NS
		Helpfulness	1.55 (0.59)	1.40 (0.57)	1.44 (0.52)	NS
Self‐reliant	Trial	Use	1.91 (0.51)	1.95 (0.15)	2.01 (0.15)	< .001 (T3 > T2)
		Helpfulness	1.37 (0.55)	2.67 (0.15)	2.79 (0.14)	< .001
	Control	Use	1.95 (0.59)	1.41 (0.55)	1.40 (0.53)	< .001(T1 > T2,T3)
		Helpfulness	1.59 (0.54)	1.11 (0.47)	1.12 (0.45)	< .001(T1 > T2,T3)
Total score	Trial	Use	1.77 (0.30)	1.76 (0.07)	1.74 (0.06)	< .05 (T2 > T3)
		Helpfulness	1.29 (0.37)	2.56 (0.11)	2.66 (0.09)	< .001
	Control	Use	1.65 (0.34)	1.66 (0.33)	1.66 (0.32)	NS
		Helpfulness	1.44 (0.37)	1.46 (0.36)	1.46 (0.34)	NS

T1, Time 1; T2, Time 2; T3, Time 3; *For significant *p* values without ranking of times, there were differences between all three times. NS, Nonsignificant.

## 4 DISCUSSION

The purpose of this study was to evaluate the efficacy of an educational program based on the transactional model of stress and coping designed to decrease perceived stress and increase healthy coping behaviors. Participants in the intervention group experienced a decrease in perceived stress over time that was significantly more than occurred among those in the control group. In addition, the use and effectiveness of problem‐focused coping styles increased and the use of emotion‐focused coping styles decreased significantly in the intervention group compared to the control group.

Other researchers have conducted studies on stress and coping styles in the people with MS. However, there are few intervention studies of this type in MS patients. Recently, Jose et al. in a systematic review of intervention studies on stress management for people with MS found that mindfulness‐based strategies significantly improved quality of life, depression, anxiety, and fatigue (Jose et al., 2016). In a study of MS patients with posttraumatic stress disorder, the efficacy of two treatments including Eye Movement Desensitization and relaxation therapy was compared in two groups of patients. Results indicated that both methods were effective in reducing stress, anxiety, depression, and improving quality of life (Carletto et al., 2016). In another randomized controlled trial, Artemiadis and colleagues examined a stress management program that included progressive muscle relaxation and breathing techniques for relapsing–remitting MS patients. They found that stress‐related symptoms decreased significantly over time with these activities (Artemiadis et al., 2012).

Our study is one of the few that have used an educational program based on a well‐known model of stress and coping (TMSC) designed to help patients cope with the stress of having MS. Other studies include the following. Pakenham examined the effects of a stress and coping model, finding that a greater emphasis on problem‐focused coping, less reliance on emotion‐focused coping, and less disability were the most important predictors of adaption (Pakenham, 1999). In an experimental study, investigators used the Fordyce Happiness model to reduce depression, stress, anxiety, and fatigue in MS patients. The focus of this model is increasing optimistic thinking and evaluating happiness in the present life, while lowering unrealistic expectations (Khayeri, Rabiei, Shamsalinia, & Masoudi, 2016). A goodness‐of‐fit model, which focused on using different approaches for controllable and uncontrollable life stressors, has also been examined in MS patients. In that study, the use of problem‐focused coping compared to meaning‐based coping styles for dealing with uncontrollable stressors was found to increase depressive and anxiety symptoms, indicating that meaning‐focused strategies may be more effective in these situations (Roubinov, Turner, & Williams, 2015). Finally, the Motivational Model of Pain Self‐Management for coping with pain is another coping with stress model. The research on this model suggests that it has considerable benefits in terms of reducing chronic pain in people with MS (Kratz, Molton, Jensen, Ehde, & Nielson, 2011). Each of these studies examined coping and stress model‐based approaches for MS patients that may help them to adapt to their disease.

The most important finding in this study was the effectiveness of the educational program based on the TMSC model in reducing perceived stress. By 1 month following the intervention, there was a considerable reduction in perceived stress, which was followed by a smaller but persistent reduction in stress by the 3‐month follow‐up. This suggests that the intervention may have its greatest impact in the short term, but over time its efficacy may reach a plateau where further programmatic activities may be needed to reinforce and maintain efficacy. In contrast to those in the intervention group, women with MS in the control group who did not receive the program experienced an increase in their stress level over time. Brown et al. followed MS patients over a period of 2 years and assessed stress levels at 3‐month intervals. They also found that stress levels increased over time among those without effective coping strategies (Brown et al., 2006). However, in a second study that compared stressful events and health status between MS patients and healthy controls during a 6‐year follow‐up, healthy controls actually reported more stressful events than patients (although this finding may have been influenced by the fact that healthy controls may have had more interactions in life activities putting them at greater risk for negative events) (Schwartz et al., 1999). The risk of disease progression in MS patients increases with increasing stress, leading to the development of a vicious cycle where stress precipitates disease progression, which in turn increases stress, which causes further disease progression, and so forth. Learning effective coping strategies (as occurred in the present program) may help to break this vicious cycle.

This study also found that use and effectiveness of problem‐focused coping strategies such as confrontive, supportive, and self‐reliant coping styles increased with the current intervention, while emotion‐focused styles such as emotive and fatalistic coping strategies tended to decrease. Although using positive‐focused coping strategies is known to be effective for controllable stressors by increasing self‐efficacy and satisfaction, emotion‐focused strategies may also be needed to deal with uncontrollable stressors that positive‐focused coping may be less effective in (Roubinov et al., 2015).

Another notable finding in this study was the increasing effectiveness of emotion‐focused coping, despite a decreasing trend for use. In other words, as participants were encouraged to use problem‐focused strategies rather than emotional‐focused ones, the helpfulness of emotion‐focused strategies for certain situations actually increased. Furthermore, in the control group, there was an increasing use of certain emotion‐focused coping strategies such as optimistic and emotive types despite their decreasing helpfulness. Thus, this suggests an urgent need to intervene among patients to guide them in selecting coping behaviors that will prove effective in dealing with MS and the stressors that accompany it.

Despite a strong study design that included an adequately powered sample size and randomization, the study also had several limitations that must be considered when interpreting the findings. First, the sample was one of convenience and all participants were volunteers, which prevents generalization of the findings to the entire universe of Iranian MS patients. A multicenter approach might have overcome this limitation to some degree. Second, a scale such as the Expanded Disability Status Scale (EDSS) to categorize participants by disability level might have been used to better tailor the intervention. Moreover, other treatments that participants were receiving such as medications and other interventions would have been helpful to better understand the clinical status of the patients. Third, the control group received only routine psychosocial care from the MS Society, leaving open the possibility that social attention and time alone may have produced the results reported here. Finally, measuring other outcomes such as anxiety, depression, and quality of life would have helped to determine the effects of the intervention on these key psychological states that MS patients often struggle, particularly depression whose assessment is strongly suggested for future studies. However, given the frail nature of the sample and the need to limit the number of questionnaire items, these outcomes were not assessed.

## 5 CONCLUSION

This study demonstrates that an educational intervention based on a transactional model of coping is beneficial in reducing perceived stress and increasing healthy coping behaviors. This model focused on positive‐focused and emotion‐focused coping behaviors known to help patients with chronic illness cope with stress. Based on these findings, the educational TMSC program appears to be one that can help many women with MS cope better with their disease. Further studies are needed to verify these findings particularly in men, and if those too are positive, then this program should be adopted throughout the country in this patient population (and possibly in those with other chronic degenerative diseases).

## CONFLICT OF INTERESTS

The Authors declare that there is no conflict of interest.

## References

[brb3810-bib-0001] AAN . (2016). Multiple sclerosis. Retrieved from https://www.aan.com/Guidelines/home/ByTopic?topicId=18. Accessed January 21, 2016.

[brb3810-bib-0002] Artemiadis, A. K. , Vervainioti, A. A. , Alexopoulos, E. C. , Rombos, A. , Anagnostouli, M. C. , & Darviri, C. (2012). Stress management and multiple sclerosis: A randomized controlled trial. Archives of Clinical Neuropsychology: The Official Journal of the National Academy of Neuropsychologists, 27(4), 406–416. 22491729 10.1093/arclin/acs039

[brb3810-bib-0003] Bergamaschi, R. , Rezzani, C. , Minguzzi, S. , Amato, M. P. , Patti, F. , Marrosu, M. G. , … DYMUS Group . (2009). Validation of the DYMUS questionnaire for the assessment of dysphagia in multiple sclerosis. Functional Neurology, 24(3), 159–162. 20018144

[brb3810-bib-0004] Brown, R. F. , Tennant, C. C. , Sharrock, M. , Hodgkinson, S. , Dunn, S. M. , & Pollard, J. D. (2006). Relationship between stress and relapse in multiple sclerosis: Part II. Direct and indirect relationships. Multiple Sclerosis, 12(4), 465–475. 16900760 10.1191/1352458506ms1296oa

[brb3810-bib-0005] Browne, P. , Chandraratna, D. , Angood, C. , Tremlett, H. , Baker, C. , Taylor, B. V. , & Thompson, A. J. (2014). Atlas of multiple sclerosis 2013: A growing global problem with widespread inequity. Neurology, 83(11), 1022–1024. 25200713 10.1212/WNL.0000000000000768 PMC4162299

[brb3810-bib-0006] Carletto, S. , Borghi, M. , Bertino, G. , Oliva, F. , Cavallo, M. , Hofmann, A. , … Ostacoli, L. (2016). Treating post‐traumatic stress disorder in patients with multiple sclerosis: A randomized controlled trial comparing the efficacy of eye movement desensitization and reprocessing and relaxation therapy. Frontiers in Psychology, 7, 526. 27148134 10.3389/fpsyg.2016.00526 PMC4838623

[brb3810-bib-0007] Cook, S. D. (2006). Handbook of multiple sclerosis, 4th ed. New York: Taylor & Francis.

[brb3810-bib-0008] Eskandarieh, S. , Heydarpour, P. , & Sahraian, M. A. (2015). Incidence and prevalence of multiple sclerosis in Iran. Multiple Sclerosis Journal, 21, 73–73.

[brb3810-bib-0009] Etemadifar, M. , Izadi, S. , Nikseresht, A. , Sharifian, M. , Sahraian, M. A. , & Nasr, Z. (2014). Estimated prevalence and incidence of multiple sclerosis in Iran. European Neurology, 72(5–6), 370–374. 25341473 10.1159/000365846

[brb3810-bib-0010] Folkman, S. , & Lazarus, R. S. (1980). An analysis of coping in a middle‐aged community sample. Journal of Health and Social Behavior, 21(3), 219–239. 7410799

[brb3810-bib-0011] Folkman, S. , & Lazarus, R. S. (1988). Ways of coping questionnaire, Research ed. Palo Alto CA: Consulting Psychologists Press.

[brb3810-bib-0012] Gruenewald, D. A. , Higginson, I. J. , Vivat, B. , Edmonds, P. , & Burman, R. E. (2004). Quality of life measures for the palliative care of people severely affected by multiple sclerosis: A systematic review. Multiple Sclerosis, 10(6), 690–704. 15584496 10.1191/1352458504ms1116rr

[brb3810-bib-0013] Harbo, H. F. , Gold, R. , & Tintore, M. (2013). Sex and gender issues in multiple sclerosis. Therapeutic Advances in Neurological Disorders, 6(4), 237–248. 23858327 10.1177/1756285613488434 PMC3707353

[brb3810-bib-0014] Izadi, S. M. , Nikseresht, A. R. M. , Poursadeghfard, M. M. , Borhanihaghighi, A. M. , & Heydari, S. T. P. (2015). Prevalence and incidence of multiple sclerosis in fars province, Southern Iran. Iranian Journal of Medical Sciences, 40(5), 390–395. 26379344 PMC4567597

[brb3810-bib-0015] Jose, A. M. S. , Oreja‐Guevara, C. , Lorenzo, S. C. , Notario, L. C. , Vega, B. R. , & Perez, C. B. (2016). Psychotherapeutic and psychosocial interventions for managing stress in multiple sclerosis: The contribution of mindfulness‐based interventions. Neurologia, 31(2), 113–120. 26385015 10.1016/j.nrl.2015.07.014

[brb3810-bib-0016] Khayeri, F. , Rabiei, L. , Shamsalinia, A. , & Masoudi, R. (2016). Effect of Fordyce Happiness Model on depression, stress, anxiety, and fatigue in patients with multiple sclerosis. Complementary Therapies in Clinical Practice, 25, 130–135. 27863602 10.1016/j.ctcp.2016.09.009

[brb3810-bib-0017] Kopke, S. , Kern, S. , Fischer, K. , Kasper, I. , Kleiter, M. , Berghoff, F. , … Heesen, C. (2012). Patient education programme on diagnosis, prognosis and early therapy for persons with early multiple sclerosis ‐ multicentre, randomised, controlled trial (ISRCTN12440282). Multiple Sclerosis Journal, 18, 475–475.

[brb3810-bib-0018] Kratz, A. L. , Molton, I. R. , Jensen, M. P. , Ehde, D. M. , & Nielson, W. R. (2011). Further evaluation of the motivational model of pain self‐management: Coping with chronic pain in multiple sclerosis. Annals of Behavioral Medicine, 41(3), 391–400. 21213092 10.1007/s12160-010-9249-6 PMC3371774

[brb3810-bib-0019] Lazarus, R. S. , & Folkman, S. (1987). Transactional theory and research on emotions and coping. European Journal of Personality, 1, 141–169.

[brb3810-bib-0020] Maroufizadeh, S. , Zareiyan, A. , & Sigari, N. (2014). Reliability and validity of Persian version of perceived stress scale (PSS‐10) in adults with asthma. Archives of Iranian Medicine, 17(5), 361–365. 24784866

[brb3810-bib-0021] McDonald, W. I. , Compston, A. , Edan, G. , Goodkin, D. , Hartung, H.‐P. , Lublin, F. D. , … Wolinsky, J. S. (2001). Recommended diagnostic criteria for multiple sclerosis: Guidelines from the International Panel on the diagnosis of multiple sclerosis. Annals of Neurology, 50(1), 121–127. 11456302 10.1002/ana.1032

[brb3810-bib-0022] Minden, S. L. , Ding, L. , Cleary, P. D. , Frankel, D. , Glanz, B. I. , Healy, B. C. , & Rintell, D. J. (2013). Improving the quality of mental health care in multiple sclerosis. Journal of the Neurological Sciences, 335(1–2), 42–47. 24183855 10.1016/j.jns.2013.08.021

[brb3810-bib-0023] Morales‐Gonzales, J. M. , Benito‐Leon, J. , Rivera‐Navarro, J. , Mitchell, A. J. , & Grp, G. S. (2004). A systematic approach to analyse health‐related quality of life in multiple sclerosis: The GEDMA study. Multiple Sclerosis, 10(1), 47–54. 14760952 10.1191/1352458504ms967oa

[brb3810-bib-0024] National MS Society . (2016). Multiple sclerosis FAQs: discover more about multiple sclerosis. Retrieved from http://www.nationalmssociety.org/What-is-MS/MS-FAQ-s. Accessed January 25, 2017.

[brb3810-bib-0025] Nejati, S. , Zahiroddin, A. , Afrookhteh, G. , Rahmani, S. , & Hoveida, S. (2015). Effect of group mindfulness‐based stress‐reduction program and conscious yoga on lifestyle, coping strategies, and systolic and diastolic blood pressures in patients with hypertension. The Journal of Tehran Heart Center, 10(3), 140–148. 26697087 PMC4685370

[brb3810-bib-0026] Noonan, C. W. , Williamson, D. M. , Henry, J. P. , Indian, R. , Lynch, S. G. , Neuberger, J. S. , … Marrie, R. A. (2010). The prevalence of multiple sclerosis in 3 us communities. Preventing Chronic Disease, 7(1), A12. 20040227 PMC2811507

[brb3810-bib-0027] Nowaczyk, N. , & Cierpialkowska, L. (2016). Coping with multiple sclerosis from the perspective of Stevan E. Hobfoll's theory of conservation of resources. Postępy Psychiatrii i Neurologii. 25(2), 111–123.

[brb3810-bib-0028] Pakenham, K. I. (1999). Adjustment to multiple sclerosis: Application of a stress and coping model. Health Psychology, 18(4), 383–392. 10431940 10.1037//0278-6133.18.4.383

[brb3810-bib-0029] Plow, M. A. , Resnik, L. , & Allen, S. M. (2009). Exploring physical activity behaviour of persons with multiple sclerosis: A qualitative pilot study. Disability and Rehabilitation, 31(20), 1652–1665. 19479491 10.1080/09638280902738375 PMC4703089

[brb3810-bib-0030] Potter, P. A. , & Perry, A. G. (2007). Basic nursing: Essentials for practice. 6th ed. St. Louis, MO: Mosby Elsevier.

[brb3810-bib-0031] de Ridder, D. , Schreurs, K. , & Bensing, J. (2000). The relative benefits of being optimistic: Optimism as a coping resource in multiple sclerosis and Parkinson's disease. British Journal of Health Psychology, 5, 141–155.

[brb3810-bib-0032] Rodriguez, M. (2008). Advances in multiple sclerosis and experimental demyelinating diseases. Berlin: Springer.

[brb3810-bib-0033] Rommer, P. S. , Suhnel, A. , Konig, N. , & Zettl, U. K. (2016). Coping with multiple sclerosis‐the role of social support. Acta Neurologica Scandinavica, 136(1), 11–16. 27620927 10.1111/ane.12673

[brb3810-bib-0034] Roubinov, D. S. , Turner, A. P. , & Williams, R. M. (2015). Coping among individuals with multiple sclerosis: Evaluating a goodness‐of‐fit model. Rehabilitation Psychology, 60(2), 162–168. 25822181 10.1037/rep0000032

[brb3810-bib-0035] Saffari, M. , Sanaeinasab, H. , Hashempour, M. , Pakpour, A. H. , Lovera, J. F. , & Al Shohaib, S. (2016). Cultural adaptation, validity, and factor structure of jalowiec coping scale in iranian women with multiple sclerosis: Which coping strategies are most common and effective? International Journal of MS Care, 19(4), 209–216. 10.7224/1537-2073.2016-042 PMC5564282 28835745

[brb3810-bib-0036] Schwartz, C. E. (1999). Teaching coping skills enhances quality of life more than peer support: Results of a randomized trial with multiple sclerosis patients. Health Psychology, 18(3), 211–220. 10357502 10.1037//0278-6133.18.3.211

[brb3810-bib-0037] Schwartz, C. E. , Foley, F. W. , Rao, S. M. , Bernardin, L. J. , Lee, H. , & Genderson, M. W. (1999). Stress and course of disease in multiple sclerosis. Behavioral Medicine, Fall. 25(3), 110–116. 10640224 10.1080/08964289909596740

[brb3810-bib-0038] Simpson, R. J. , McLean, G. , Guthrie, B. , Mair, F. , & Mercer, S. W. (2014). Physical and mental health comorbidity is common in people with multiple sclerosis: Nationally representative cross‐sectional population database analysis. BMC Neurology, 14, 128. 24925477 10.1186/1471-2377-14-128 PMC4064287

[brb3810-bib-0039] Solaro, C. , Rezzani, C. , Trabucco, E. , Amato, M. P. , Zipoli, V. , Portaccio, E. , … Bergamaschi, R. (2013). Prevalence of patient‐reported dysphagia in multiple sclerosis patients: An Italian multicenter study (using the DYMUS questionnaire). Journal of the Neurological Sciences, 331(1–2), 94–97. 23747002 10.1016/j.jns.2013.05.020

[brb3810-bib-0040] Thompson, A. J. , Rompani, P. , Dua, T. , Douglas, I. , Battaglia, M. , & Porter, B. (2008). WHO/MSIF: Atlas of MS: multiple sclerosis resources across the world. Multiple Sclerosis, 14, S153–S153.

[brb3810-bib-0041] Weiten, W. (2009). Psychology applied to modern life: Adjustment in the 21st century, 9th ed. Belmont, CA: Wadsworth Cengage Learning.

[brb3810-bib-0042] Zeidner, M. , & Endler, N. S. (1996). Handbook of coping: Theory, research, applications. New York: Wiley.

